# A computerized version of the Short Form of the Face-Name Associative Memory Exam (FACEmemory®) for the early detection of Alzheimer’s disease

**DOI:** 10.1186/s13195-020-00594-6

**Published:** 2020-03-16

**Authors:** Montserrat Alegret, Nathalia Muñoz, Natalia Roberto, Dorene M. Rentz, Sergi Valero, Silvia Gil, Marta Marquié, Isabel Hernández, Catalina Riveros, Angela Sanabria, Alba Perez-Cordon, Ana Espinosa, Gemma Ortega, Ana Mauleón, Carla Abdelnour, Maitee Rosende-Roca, Kathryn V. Papp, Adela Orellana, Alba Benaque, Lluís Tarraga, Agustín Ruiz, Mercè Boada

**Affiliations:** 1 Research Center and Memory Clinic, Fundació ACE, Institut Català de Neurociències Aplicades, Universitat Internacional de Catalunya, Gran Via de Carles III, 85 bis, 08028 Barcelona, Spain; 2 Networking Research Center on Neurodegenerative Diseases (CIBERNED), Instituto de Salud Carlos III, Madrid, Spain; 3 Department of Neurology, Center for Alzheimer Research and Treatment, Brigham and Women’s Hospital, Boston, MA USA; 4 Department of Neurology, Massachusetts General Hospital, Boston, MA USA

**Keywords:** Memory, Early detection, New technologies, Alzheimer’s disease, Computerized assessment, Self-administration, Cognitive impairment, Biomarkers, Cerebrospinal fluid

## Abstract

**Background:**

Computerized neuropsychological tests for early detection of Alzheimer’s disease (AD) have attracted increasing interest. Memory for faces and proper names is a complex task because its association is arbitrary. It implicates associative occipito-temporal cerebral regions, which are disrupted in AD. The short form of the Face-Name Associative Memory Exam (FNAME-12), developed to detect preclinical and prodromal AD, asks individuals to learn the names and occupations associated with 12 faces. The current work advances this field by using voice recognition and touchscreen response format. The purpose of this study is to create the first self-administered episodic memory test, FACEmemory®, by adapting the FNAME-12 for tablet use with voice recognition, touchscreen answers, and automatic scoring. The test was minimally supervised by a psychologist to avoid technological problems during execution and scored manually to assess the reliability of the automatic scoring. The aims of the present study were (1) to determine whether FACEmemory® is a sensitive tool for the detection of cognitive impairment, (2) to examine whether performances on FACEmemory® are correlated with those on the S-FNAME (paper-and-pencil version with 16 images), and (3) to determine whether performances on FACEmemory® are related to AD biomarkers in the cerebrospinal fluid (CSF) (Aβ42, p-tau, and Aβ42/p-tau ratio).

**Methods:**

FACEmemory® was completed by 154 cognitively healthy (CH) individuals and 122 subjects with mild cognitive impairment, of whom 61 were non-amnestic (naMCI) and 61 amnestic (aMCI). A subsample of 65 individuals completed the S-FNAME, and 65 subjects received lumbar punctures.

**Results:**

Performance on FACEmemory® was progressively worse from CH to the naMCI and aMCI groups. A cutoff of 31.5 in total FACEmemory® obtained 80.5% and 80.3% sensitivity and specificity values, respectively, for discriminating between CH and aMCI. Automatically corrected FACEmemory® scores were highly correlated with the manually corrected ones. FACEmemory® scores and AD CSF biomarker levels were significantly correlated as well, mainly in the aMCI group.

**Conclusions:**

FACEmemory® may be a promising memory prescreening tool for detecting subtle memory deficits related to AD. Our findings suggest FACEmemory® performance provides a useful gradation of impairment from normal aging to aMCI, and it is related to CSF AD biomarkers.

## Background

One of the endophenotypes proposed for Alzheimer’s disease (AD) is episodic memory tests [1]. Cognitive approaches conceptualizing face-name associative memory have demonstrated that associating unfamiliar faces with proper names is a task more complex than other visual memory tests because this is an arbitrary association [2]. It implicates associative occipito-temporal cerebral regions with extensive connections to cortical areas, which is the specific neural function disrupted in AD [3, 4]. The Face-Name Associative Memory Exam (FNAME) [4] was developed to detect preclinical AD. The FNAME is an associative episodic memory test that has been validated in American [5] and Spanish [6] populations. Patients with mild cognitive impairment (MCI) have lower performances on the FNAME than cognitively healthy (CH) individuals [7]. Moreover, low scores on the original and Spanish (S-FNAME) versions of the FNAME have been found to be associated with biomarker evidence of increased cerebral amyloid burden, quantified by positron emission tomography in healthy elderly [4, 8].

A shortened and optimized version of the FNAME (FNAME-12), whose scores are strongly correlated with those on the original FNAME (*r* = 0.77), was recently created [9] and subsequently validated in American [9], Latino American [10], and Greek populations [11]. In contrast with the original FNAME, the FNAME-12 comprises fewer stimuli, an additional learning trial, and a delayed memory recognition task. This shortened version can be used not only in preclinical AD, but also in prodromal AD, making it possible to discriminate between normal aging and MCI [9], with the latter group converting to dementia in most cases [12, 13]. However, the FNAME-12 retains the main features of the original FNAME: a paired associative learning paradigm and its ecological validity to test the difficulty in retrieving newly learned face-name pairs frequently complained among elderly people. The FNAME-12 was modified to include an additional learning trial and a recognition task.

In the last decade, computerized neuropsychological tests have generated increasing interest in clinical practice contexts [14], especially for early detection of AD. For this reason, the FNAME was recently self-administered, without supervision, to 49 CH individuals aged between 60 and 87 years, as part of a Computerized Cognitive Composite for Preclinical Alzheimer’s Disease (C3-PAD) test with a touchscreen. A majority completed the test successfully [15].

Other computerized neuropsychological tools, such as the Cambridge Neuropsychological Test Automated Battery (CANTAB) [16], comprise memory tests that can be administered with a touchscreen tablet. A bad performance on its episodic memory subtest, the Paired Associates Learning (PAL), has been found to be associated with AD biomarkers, such as reduced hippocampal volume and increased levels of total tau (t-tau) and phosphorylated tau (p-tau) in cerebrospinal fluid (CSF) [1]. However, to the best of our knowledge, there are no verbal episodic memory tests that can be self-administered on a tablet computer with voice recognition and a touchscreen and then automatically scored, registered, and saved in a database.

For the purpose of the present study in Fundació ACE, we developed a self-administered computerized version of the FNAME-12 (named FACEmemory®) with images, names, and occupations representative of the Spanish population. The test was administered using a tablet computer with voice recognition and a touchscreen, enabling us to immediately score and register the results in a database, as a tool for the early detection of cognitive impairment and AD. We hypothesized that FACEmemory® would identify individuals with early cognitive impairment at risk of developing AD, as well as providing immediate scoring and ensuring maximum standardization and reliability.

The main objectives of the present study were the following: (1) to determine whether FACEmemory® was a sensitive tool for the detection of cognitive impairment, (2) to assess whether performances on FACEmemory® were correlated with those on the S-FNAME (paper-and-pencil version with 16 images), and (3) to determine whether performances on FACEmemory® were related to AD biomarkers in CSF (amyloid beta 42 [Aβ42], p-tau, and Aβ42/p-tau ratio).

## Methods

### Participants

A sample of 276 individuals older than 50 years participated in this study: 154 CH individuals and 122 subjects diagnosed with MCI, of whom 61 were non-amnestic (naMCI) and 61 amnestic (aMCI). All of them were evaluated at Fundació ACE, *Institut Català de Neurociències Aplicades* (Barcelona, Spain), a non-profit Alzheimer’s institution that provides diagnostic, treatment, and patient management services to the Catalan Public Health Service [17]. All participants underwent a complete neuropsychological assessment using the Neuropsychological Battery of Fundació ACE (NBACE), whose normative data and cutoff scores have been reported elsewhere [18]; a neurological history and examination; a semi-structured psychosocial interview, including a functionality assessment by the Blessed Dementia Rating Scale (BDRS) [19, 20]; and a diagnosis of their cognitive status by the clinical team from the Memory Unit at a daily consensus conference [17]. The sample comprised 64 participants enrolled in the Fundació ACE Healthy Brain Initiative (FACEHBI) study [21], 58 participants in the BIOFACE cohort, 42 individuals recruited from the Open House Initiative (OHI) [22], and 112 subjects evaluated at Fundació ACE’s Memory Unit.

The inclusion criteria for the whole sample were the following: age over 50, an educational level of at least elementary school (in order to ensure the correct understanding of the FACEmemory® instructions), a clinical diagnosis of CH or MCI [17], and completion of FACEmemory®.

The clinical diagnoses for the CH group were as follows: absence of objective cognitive impairment, with average or above-average scores on NBACE [18, 23]; normal general cognition (Mini-Mental State Examination (MMSE) score ≥ 27) [24, 25]; a Clinical Dementia Rating (CDR) [26] of 0; and no history of functional impairment due to declining cognition, with a score below 4 on the BDRS [19, 20].

The clinical diagnosis for the MCI group were as follows: subjective cognitive complaints, essentially preserved general cognitive function (MMSE score ≥ 24) [24, 25], preserved performance in activities of daily living (BDRS < 4) [19, 20], absence of dementia; a CDR [26] of 0.5, an objectively measurable impairment in memory or another cognitive function (aMCI or naMCI) [12, 27], and the absence of prescribed symptomatic treatment for dementia (i.e., acetylcholinesterase inhibitors or memantine).

Participants were not required to have previous knowledge of tablet computer use. To ensure the correct understanding of the FACEmemory® instructions and the completion of the test, exclusion criteria included an educational level below elementary school and significant auditory or visual abnormalities, such as glaucoma, cataracts, or severe aphasia.

As mentioned above, a subsample of 65 individuals was also administered the S-FNAME (paper-and-pencil version with 16 items). That is, they completed FACEmemory® and S-FNAME on different days (no more than 2 months apart).

Finally, 65 subjects underwent lumbar puncture (LP) to measure AD biomarkers in CSF.

In collaboration with Dr. Rentz’s team, we transformed the original paper-and-pencil FNAME-12 [9] into a self-administered computerized version (named FACEmemory®) with images, names, and occupations representative of the Spanish population. The test was administered using a tablet computer with voice recognition and touchscreen, enabling us to immediately score and register the results anonymously in a database. The scores of all variables ranged from 1 to 12, and the total FACEmemory® scores ranged from 1 to 96.

### The FACEmemory® procedure

All subjects completed FACEmemory® on a tablet with voice recognition, a touchscreen, and scoring registration in Fundació ACE. The temporal sequence of FACEmemory® was the following: two learning trials, a short-term memory task, and a long-term memory task that included face, name, and occupation recognition. The total test duration was approximately 30 min.

After the study, participants completed a satisfaction survey to indicate their level of satisfaction with the test. The survey consisted of 5 questions and required the participants to score their satisfaction from 1 to 5 (1 being the worst score), to choose what aspect of the test they disliked the most, to indicate whether they would recommend the test to family or friends, to select what aspect they liked the most, and to mention whether they would like to repeat the test in the future.

FACEmemory® was self-administered with minimal supervision from a psychologist (who only addressed technological issues). The psychologist simultaneously scored the test manually to determine whether the automatic scoring matched the hand scoring.

**Fig. 1:**
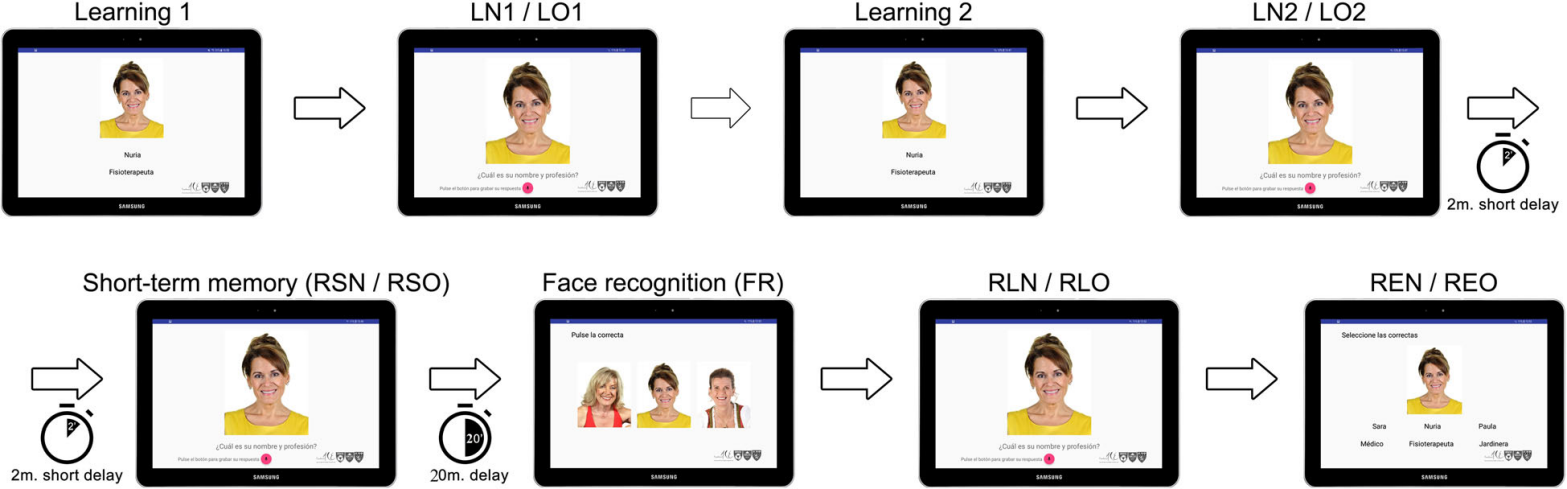
The FACEmemory® procedure. The participants underwent two exposures (learning 1 and learning 2) to all the 12 face, name, and occupation groupings. Following each exposure, they were asked to give the name (LN1/LN2) and the occupation (LO1/LO2) associated with each face. After a 2-min delay, they were asked to provide the name (RSN) and the occupation (RSO) associated with each face. Following a 20-min delay, they were asked to identify the previously learned face from two pictures (FR). They were again asked to give the name (RLN) and the occupation (RLO) associated with each face. The participants were asked to select the name and/or occupation associated with the face from among three items (REN/REO)

As shown in Fig. 1, the first learning trial consisted of a total of 12 faces, each one associated with a name and an occupation that appeared beneath it for 8 s. The second learning trial was identical, except that the faces appeared in a different order. The participants were instructed to read the name and occupation appearing beneath each face aloud and to try to remember it. Then, the application asked the participants to press the red microphone button and to say the name (LN1/LN2) and occupation (LO1/LO2) they remembered as being associated with each face. If they did not remember anything, they were allowed to say “I don’t know.”

For the evaluation of short-term memory, 2 min after the second learning trial, the application again asked the participants to say the name (RSN) and occupation (RSO) they remembered as being associated with each face, but if they did not remember anything, they could say “I don’t know.” They had to press the red microphone button and to answer.

Finally, 20 min after the second learning trial (LN2/LO2), the application started the long-term memory assessment, which involved free recall and recognition tasks. First, the participants were instructed to recognize, out of 3 faces, the face that had appeared in the first learning trial and to touch it (FR). Then, the correct face appeared, and the application asked the participants to say the name (RLN) and occupation (RLO) they remembered for each face. After each answer, a screen appeared, showing the right face and 2 rows beneath it. Each row had 3 name and 3 occupation options. The participants were instructed to touch the name (REN) and occupation (REO) they remembered as being associated with that face.

The application registered the touched responses and the voice of the participants automatically and entered the scores into a database anonymously.

### Application development

The application was developed with Android language and using Google Books for voice recognition and interactions with the device. Google voice recognition libraries were used, as they are the most complete and because they allow the use of different languages.

During development, screens were constantly checked to ensure they were clear, so that the user’s attention would not be distracted while they were learning the data presented and so that the test would be as reliable as possible. The application has been designed so that when the user interacts with it using voice commands, the elements that appear on the screen are minimal. The FACEmemory® application is linked to a control panel or back end, from which the saved data from all the tests performed can be viewed and extracted, and filtered by date or test. Access to the back end requires a username and password.

One of the most important features of the FACEmemory® application is that it keeps the user’s data completely anonymous for better protection. Despite this anonymity, the application is configured so that a user can perform multiple tests at different times. When a participant finishes FACEmemory®, the application generates a code Fundació ACE saves on a separate system that complies with the General Data Protection Regulation (GDPR) and that connects the user to the test carried out. Therefore, there are data saved from individual tests or tests related to others saved in the system, but no personal data, thus complying with the GDPR at all times. Security and data encryption measures have been also applied to communications between system elements, to make both the tablet application and the back office safe and robust.

In summary, the FACEmemory® application allows the user to focus their attention on the test, as well as allowing Fundació ACE to manage the data in a simple and anonymous way.

### Lumbar puncture and cerebrospinal fluid collection

This protocol followed the consensus recommendations established by the Alzheimer’s Biomarkers Standardization Initiative [28]. Briefly, those participants who agreed to undergo a LP in Fundació ACE had the procedure performed by an experienced neurologist, with the patients in a seated position and under fasting conditions. After applying local anesthesia (1% mepivacaine) subcutaneously, the neurologist obtained CSF by LP in the intervertebral space of L3–L4. The fluid was collected passively in two 10-ml polypropylene tubes (Stardest Ref. 62610018). The first tube was analyzed externally for basic biochemistry (glucose, total proteins, proteinogram, and cell type and number). The second tube was centrifuged (2000×*g* 10 min at 4 °C), and the fluid was aliquoted into polypropylene tubes (Stardest Ref. 72694007) and stored at − 80 °C until analysis. The time delay between CSF collection and storage was less than 2 h.

On the day of the analysis, the aliquots were thawed at room temperature and vortexed for 5–10 s to determine AD biomarkers in CSF [29]. One aliquot/patient was used to determine the concentrations of Aβ1-42, t-tau, and p181-tau using the commercially available enzyme-linked immunosorbent assays (Innotest, Fujirebio Europe) at the Research Laboratory of Fundació ACE.

### Statistical analysis

Statistical analyses were performed using SPSS (version 20.0; SPSS Inc., Chicago, IL). Univariate analysis of variance (ANOVA) with post hoc comparisons (Bonferroni) was used to compare sociodemographic data between the CH, naMCI, and aMCI groups. An ANOVA adjusted by years of formal education, age, and sex (ANCOVA) was carried out to compare FACEmemory® scores between the CH, naMCI, and aMCI groups.

Logistic regression analyses were carried out to search for discrimination indexes, in which the diagnostic comparisons between CH/MCI, CH/aMCI, and CH/naMCI individually were the dependent variables, and the total FACEmemory® score was the independent variable. Moreover, diagnostic sensitivities, specificities, and total FACEmemory® cutoff, as measured by the analysis of the receiver operating characteristic (ROC) curve, were obtained for the purpose of discriminating between CH/MCI and CH/aMCI. Cutoff points for the whole sample were established by calculating the sensitivity and specificity for the total FACEmemory® score. ROC analysis was used to calculate the optimal cutoff value between CH and the MCI and aMCI groups. Moreover, to calculate the sensitivity and specificity of the cutoff score, an area under the curve (AUC) analysis was also carried out and yielded a confidence interval of 95%. Our goal was to obtain an AUC greater than 0.75 for the total FACEmemory® variable and for the sensitivity and specificity values.

To ensure the reliability of the automatic scoring, Pearson’s correlation analyses were carried out between hand and automatic (obtained from voice recognition/touchscreen) scorings of the FACEmemory® test. We also correlated performances on FACEmemory® with those on the S-FNAME (paper-and-pencil version with 16 items) to examine convergent validity. Finally, partial correlation analyses were performed between the FACEmemory® scores and CSF AD biomarkers, adjusting for age, sex, and education.

## Results

**Fig. 2:**
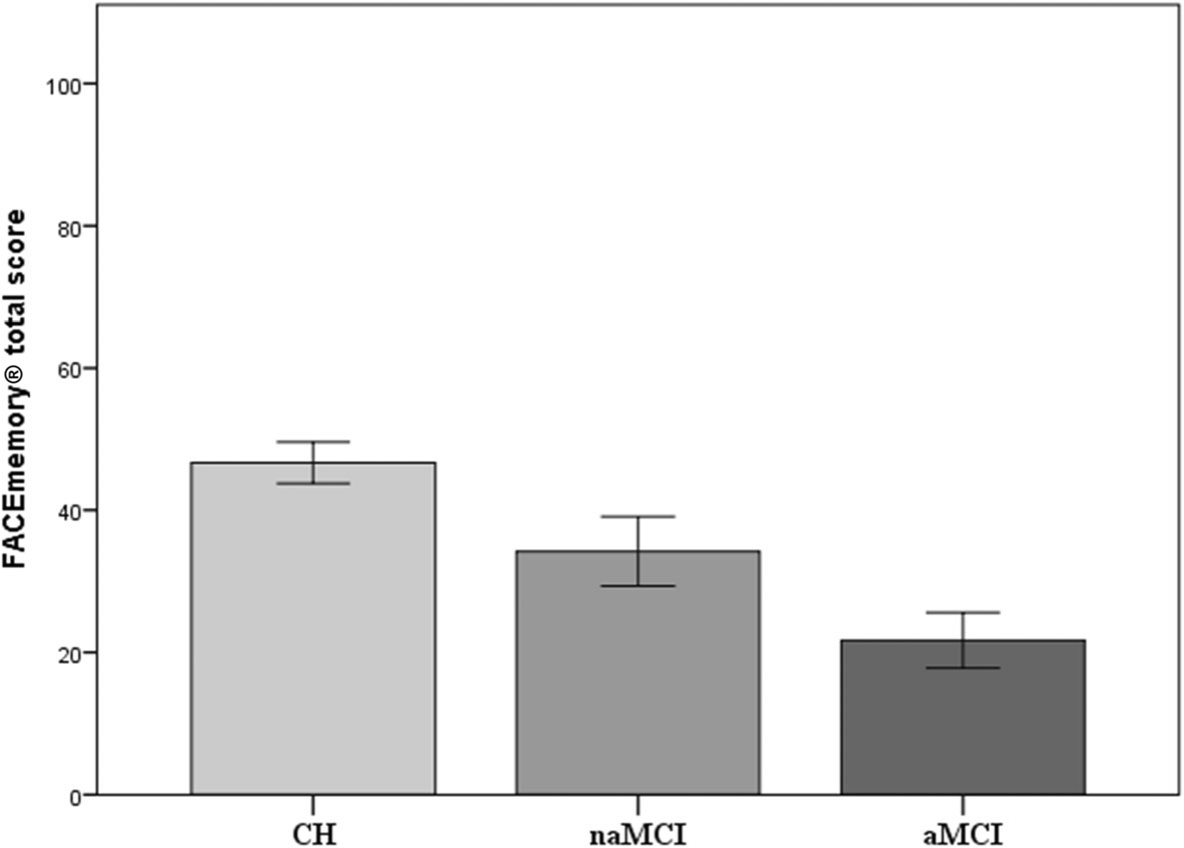
Performance on FACEmemory® in the cognitively healthy, non-amnestic MCI, and amnestic MCI groups

The CH, naMCI, and aMCI groups were statistically similar in terms of age and sex. However, the participants from the CH group had a significantly higher education than those from the MCI group (Table 1). An ANCOVA, adjusted by education, showed that the total FACEmemory® score significantly differed between the groups, with the aMCI group obtaining worse scores (Fig. 2). As detailed in Table 2, all FACEmemory® subscores differed significantly between the groups.

**Table 1:** Demographic and clinical characteristics of the participants

	CH	naMCI	aMCI	Statistics
*n*	154	61	61	
Sex, *n* (%) male	61 (39.61)	19 (31.15)	23 (37.70)	1.34_1_
Age, years (mean/SD)	67.98 (7.92)	65.98 (8.70)	67.74 (7.93)	1.36_2_
Education, years (mean/SD)	12.62 (4.18)	10.70 (4.01)	10.20 (4.04)	9.67_2_***
MMSE (mean/SD)	29.29 (0.89)	28.25 (1.58)	27.21 (1.77)	54.56_2_***

*CH* cognitively healthy, *naMCI* non-amnestic mild cognitive impairment, *aMCI* amnestic mild cognitive impairment, *SD* standard deviation, _*1*_*χ*^2^, _*2*_ F

***p < 0.001

**Table 2:** Differences between the groups on FACEmemory® total and subtest scores

Test	CH, mean (SE)	naMCI, mean (SE)	aMCI, Mean (SE)	*F*	Post hoc	*R*^2^
LN1+2	6.58 (4.79)	3.88 (4.03)	1.70 (2.66)	28.341***	All	0.24
LO1+2	14.13 (4.38)	11.85 (5.43)	8.69 (5.44)	23.073***	All	0.20
RSN	4.69 (3.31)	2.66 (3.01)	1.18 (1.70)	30.079***	All	0.25
RSO	8.65 (2.43)	7.44 (2.85)	5.13 (3.09)	29.891***	All	0.25
FR	11.96 (0.19)	11.93 (0.31)	11.54 (0.96)	11.044***	^ab^	0.11
RLN	4.42 (3.24)	2.51 (2.78)	1.02 (1.53)	32.180***	All	0.26
RLO	8.05 (2.70)	6.66 (3.31)	4.38 (3.21)	27.657***	All	0.23
REN	9.58 (2.02)	8.49 (2.64)	7.05 (2.53)	24.461***	All	0.21
REO	11.60 (0.80)	10.98 (1.52)	9.89 (2.17)	25.665***	All	0.22
Total score	46.51 (18.01)	34.98 (18.81)	22.10 (15.08)	38.511***	All	0.30

*CH* cognitively healthy, *naMCI* non-amnestic mild cognitive impairment, *aMCI* amnestic mild cognitive impairment, *SE* standard error, *All* all scores significantly differed between the CH, naMCI, and aMCI groups

***p < 0.001

^a^Scores differed significantly between the CH and aMCI groups

^b^Scores differed significantly between the aMCI and naMCI groups

With regard to discrimination indexes, logistic regression analyses, controlling for education, showed that the total FACEmemory® score significantly discriminated between the CH and MCI (naMCI + aMCI), naMCI, and aMCI groups. The best sensitivity and specificity values were obtained when the CH and aMCI groups were contrasted (82% and 80%, respectively) (Table 3).

**Table 3:** Results of the logistic regression analyses of the FACEmemory® groups

Groups	Wald	*p* value	OR	95% CI
CH-MCI^a^	36.05	0.001	0.95	0.93–0.97
CH-naMCI^a^	10.24	0.001	0.97	0.95–0.99
CH-aMCI^a^	39.73	0.001	0.92	0.89–0.94

*CH* cognitively healthy, *MCI* mild cognitive impairment (naMCI + aMCI), *naMCI* non-amnestic MCI, *aMCI* amnestic MCI

^a^Reference category: CH

The total FACEmemory® cutoffs were reported after we ensured that the AUC was greater than 0.75 and identified the cutoff score that yielded approximately equal sensitivity and specificity values (see Table 4).

**Table 4:** Cutoff of the total FACEmemory® score

Groups	ROC	95% CI	Sensitivity (%)	Specificity (%)	Cutoff
CH-MCI^a^	0.77	0.71–0.82	73.4	72.1	36.5
CH-naMCI^a^	0.68	0.60–0.76	60.0	66.0	39.5
CH-aMCI^a^	0.85	0.80–0.91	80.5	80.0	31.5

Manual and automatic scores of the total FACEmemory® were found to be highly correlated (*r* = 0.98, *p* < 0.001) in the whole sample. A significant correlation (*r* = 0.69, *p* < 0.0001) between both tests was found in the subsample of 65 subjects who completed FACEmemory® and the S-FNAME.

Participants who underwent a LP (*n* = 65) (16 CH, 28 naMCI, and 21 aMCI) were significantly older than those who did not (mean/SD = 63.89/6.56, 68.59/8.23, respectively, *t* = 4.74, *p* < 0.001), but both groups were similar in terms of sex (37% and 40% men, respectively, chi square = 0.19, *p* = 0.66), education (mean/SD = 11.65/4.18, 11.66/4.27, respectively; *t* = − 0.03, *p* = 0.98), and FACEmemory® performance (mean/SD = 36.60/21.67, 39.20/19.65, respectively; *t* = 0.91, *p* = 0.36). For this reason, partial correlations between total FACEmemory® scores and AD biomarker levels in CSF were controlled for age. In the whole sample, total FACEmemory® scores were significantly correlated with Aβ42 (*r* = 0.33), Aβ42/p-tau ratio (*r* = 0.50), t-tau (*r* = − 0.44), and p-tau (*r* = − 0.43) levels. However, when analyses were stratified by the diagnostic group, the highest correlation values were obtained in the aMCI group, between FACEmemory® performance and Aβ42 (*r* = 0.62) and Aβ42/p-tau ratio (*r* = 0.68) levels. By contrast, no statistically significant correlations were found between FACEmemory® performance and CSF biomarker levels in the CH group (for details, see Table 5).

**Table 5:** Partial correlation values between total scores on FACEmemory® and AD biomarker levels in CSF, covariated by age

FACEmemory®	Whole sample, *n* = 65	CH group, *n* = 16	naMCI group, *n* = 28	aMCI group, *n* = 21
Aβ42	0.33*	0.14	0.06	0.62**
t-tau	− 0.44***	0.08	− 0.46*	− 0.44*
p-tau	− 0.43***	0.01	− 0.49*	− 0.37
Aβ42/p-tau	0.50***	0.04	0.50*	0.68***

*CH* cognitively healthy, *naMCI* non-amnestic mild cognitive impairment, *aMCI* amnestic mild cognitive impairment, *Aβ42* amyloid beta 42, *t-tau* total tau, *p-tau* phosphorylated tau

*p < 0.05; **p < 0.005; ***p < 0.001

The results of the satisfaction survey showed that 65.2% of the participants (*n* = 180) scored 5 (*excellent*), 23.2% (*n* = 64) 4 (*very good*), 9.4% (*n* = 26) 3 (*good*), 1.8% (*n* = 5) 2 (*medium*), and 0.4% (only one) 1 (*bad*). The mean satisfaction score was 4.51 (SD = 0.77) points, and there were no significant differences among the CH (mean/SD, 4.58/0.63), aMCI (4.30/0.99), and naMCI (4.50/0.99) groups; *F*(2, 217) = 2.91, *p* = 0.06. When participants were asked whether they would recommend the test to a friend or a relative, most of them (94.2%) answered “yes” and a few (5.8%) answered “no”; most of them (97%) confirmed they would be willing to repeat the test in the future.

## Discussion

The results of the present study reveal that the self-administered computerized FACEmemory®, conducted under minimal supervision to avoid technological problems, might be a useful tool for the early detection of AD. In this study, its sensitivity and specificity values were adequate, confirming its capacity to discriminate between CH and MCI, mainly the amnestic type. As expected, performances on the computerized FACEmemory® were related to the manual scoring of FACEmemory® and the Spanish version of its original paper-and-pencil test (S-FNAME) [4, 6], confirming that it is an associative episodic memory tool with highly reliable scoring. Moreover, performances on FACEmemory® were related to CSF biomarker levels, mainly in the aMCI group, indicating that FACEmemory® is related to AD biomarkers, such as Aβ42 and Aβ42/p-tau ratio.

The novelty of the present study is that FACEmemory® is the first self-administered verbal episodic memory test application with voice recognition and touchscreen. These features ensure the standardization of the administration and scoring, and registration of performance data related to, a sensitive tool for determining memory impairment. The test is correlated with CSF markers of AD neurodegeneration.

In the search for new memory endophenotypes of AD, the present study has examined a computerized adaptation of a face-name task [9]. Attention to these endophenotypes has been relatively lacking compared to the attention given to biomarkers. AD is a specific disease of a neuroplasticity mechanism related to the fundamental role of the amyloid precursor protein in episodic memory encoding. AD-related biomarkers, including neuroimaging, CSF, or plasma, whether related to Aβ, microtubule-associated protein tau, or another biochemical abnormality, are secondary to the attack of AD pathology on an episodic memory-encoding mechanism in the brain [30]. Accordingly, measures of episodic memory, the function most vulnerable to AD’s pathological processes, are of prime importance for the early detection of AD-related dysfunction and follow-up of the disease progression [1, 12].

The FNAME was created [4] in an attempt to detect subtle memory deficits in individuals with preclinical AD and validated in its original version [5], in Greek [11], in Latino American [10], and in Spanish [6] populations. Consistent with the findings of Rentz et al. [4, 5], our team found that the S-FNAME is sensitive to episodic memory [6, 7] and related to amyloid burden, as measured by ^18^F-florbetaben positron emission tomography in healthy elderly [7]. Since faces represent information encoded by the non-dominant (usually right) hemisphere and names (and occupations) are information encoded by the language areas of the brain, the dominant (usually left) hemisphere, their combination requires communication between the two hemispheres across the corpus callosum [3]. Thus, this tool provides a strong test of functions highly vulnerable to the AD process in both hemispheres of the brain.

In contrast to paper-and-pencil tests, computerized neuropsychological tests have benefits that make them suitable for early detection of cognitive changes in the elderly, such as minimization of ceiling effects through management of test difficulty, standardized administration and item presentation, accurate response recordings, prompt and automated scoring, and reduced administration costs [31, 32]. Moreover, in contrast to FACEmemory®, computerized memory tests mostly depend on recognition rather than free recall, the measure most sensitive to memory decline in cognitively healthy and MCI individuals [12, 33, 34]. The high correlation values reached between automatic and manual FACEmemory® scores (*r* = 0.98) confirmed the reliability of FACEmemory® automated scoring.

Since the S-FNAME was not recommended to be administered to individuals with MCI as it was too challenging for them [35], the FNAME-12 was created. This is a shortened version with more learning trials and a delayed recognition task [9]. Consistent with Papp et al.’s [9] findings, in which the paper-and-pencil FNAME-12 was highly correlated (*r* = 0.77) with the original S-FNAME (paper-and-pencil version with 16 faces), we found the computerized FACEmemory® scores were significantly correlated with the paper-and-pencil S-FNAME (*r* = 0.69), confirming that the computerized version is sensitive to complex associative memory, as expected. The reason the correlation values were not higher is because the original S-FNAME and FACEmemory® have some differences, such as the administration form (paper-and-pencil vs computerized), the number of faces (16 vs 12), and the target population (preclinical vs preclinical and prodromal AD). These findings parallel previous studies demonstrating that S-FNAME [4, 6, 7, 36] and its original paper-and-pencil short form, FNAME-12 [9], are complex and ecological tests sensitive to episodic memory. FACEmemory® also has ecological validity, given that associating names and occupations with new faces is an everyday event and at the same time, performing poorly at it is a common complaint among older adults [9].

Several studies have reported memory tests (e.g., the Free and Cued Reminding Test [37], the Word List from the Wechsler Memory Scale-III [23], the computerized CANTAB PAL [38], and CogState’s One Card Learning subtest [39, 40]) as valid tools for detecting MCI, a phenotype with an increased risk of conversion to dementia, mainly of the AD type [12, 13]. In this line, consistent with the paper-and-pencil FNAME-12 results [9], performances on the first self-administered verbal memory test, FACEmemory®, got worse from the CH to the naMCI to the aMCI groups, providing a useful gradation of impairment from normal aging to aMCI. Moreover, our results showed that FACEmemory® reached adequate sensitivity and specificity values to discriminate between CH and MCI subjects, mainly the aMCI. Thus, FACEmemory® may be a suitable test for detecting MCI, mainly the amnestic type. While a sensitivity of 80.5% and a specificity of 80% might not compare well to some other sensitivity/specificity metrics, FACEmemory® is still able to detect subtle cognitive changes, distinguishing between CH and MCI individuals.

A consistent path was observed throughout the study. The FACEmemory® scores of patients in the naMCI group were consistently better than those of the aMCI group and consistently worse than those of the CH group. While this can be seen as validation evidence that FACEmemory® primarily relies on episodic memory, it also implies that the test taps into other cognitive domains, such as executive functions. Several studies have reported that the correct learning and recall of face-name pairs require coordinated activity in a distributed memory network, including the hippocampus and prefrontal cortex, which are involved in encoding and retrieval of associative memories [41, 42].

Finally, lower scores on FACEmemory® were found to be related to higher evidence of CSF AD biomarkers. That is, total FACEmemory® scores were found to be significantly and inversely correlated with CSF Aβ42, mainly in the aMCI group, the group with the highest and most imminent risk of conversion to dementia [12, 13]. This result is consistent with those of other studies reporting a link between performances on episodic memory tests, such as the original FNAME [4], the S-FNAME [8], the PAL CANTAB [1], or the Ancient Farming Equipment Test [43], and biomarkers of AD pathology, either PET imaging or CSF. Moreover, our results are consistent with the findings showing that CSF tau levels are more related to cognitive functioning, mainly memory, than CSF Aβ levels [43–48]. Additionally, the Aβ/p-tau ratio, which includes both AD biomarkers, was the CSF measurement that best correlated with performance on FACEmemory®, reinforcing the hypothesis that this test is a diagnostic tool that reflects AD pathology changes [49].

Finally, the feasibility of FACEmemory® was supported by its capacity to obtain valid data from all the participants, including those with MCI. That is, all the participants completed the test and gave a favorable opinion of the tool, with a mean satisfaction score of 4.51 points (5 being the best judgment), independently of their diagnosis group. Most of them (96.7%) confirmed they would be willing to repeat the test in the future. Like those of studies involving other computerized tests, such as CogState [40], CANTAB [38], and C3-PAD [15], our results reinforce the fact that computerized tests are feasible, suitable, and valid in elderly populations, not only in clinical practice but also for clinical trials.

A limitation of this work is that it is a single-center study. Therefore, this design does not represent the general population. However, this is the first step. We expect that later on FACEmemory® will be extended to the rest of the Spanish-speaking population, fully self-administered, and translated into other languages, so that it can be administered to all out-patient clinics, particularly those in isolated areas with fewer resources. Further longitudinal studies will be needed to determine whether lower baseline scores on FACEmemory® are related to an increased risk of developing AD.

## Conclusion

The present study demonstrated that the computerized memory test FACEmemory® may be a suitable test for detecting early cognitive impairment, mainly of the amnestic type. The test was well accepted by participants. Their test performance was found to worsen from normal aging to the naMCI to aMCI groups and was related to AD CSF biomarkers. A computerized test like this ensures standardized administration and scoring, and it might be a helpful memory prescreening tool.

## Data Availability

Data used can be requested through the corresponding author.

## Abbreviations

Aβ42: Amyloid beta 42
AD: Alzheimer’s disease
aMCI: Amnestic mild cognitive impairment
ANCOVA: Analysis of covariance
ANOVA: Univariate analysis of variance
AUC: Area under the curve
BDRS: Blessed Dementia Rating Scale
C3-PAD: Computerized Cognitive Composite for Preclinical Alzheimer’s Disease
CANTAB: Cambridge Neuropsychological Test Automated Battery
CDR: Clinical Dementia Rating
CH: Cognitively healthy
CSF: Cerebrospinal fluid
FACEHBI: Fundació ACE Healthy Brain Initiative
FACEmemory®: Computerized version of the Short Form of the Face-Name Associative Memory Exam (by tablet). Copyright© 2017
FNAME: Face-Name Associative Memory Exam
FNAME-12: The short form of the Face-Name Associative Memory Exam
FR: Face recognition score
GDPR: General Data Protection Regulation
LN1: Names recalled in learning 1
LN2: Names recalled in learning 2
LO1: Occupations recalled in learning 1
LO2: Occupations recalled in learning 2
LP: Lumbar puncture
NBACE: Neuropsychological Battery of Fundació ACE
MCI: Mild cognitive impairment
MMSE: Mini-Mental State Examination
naMCI: Non-amnestic mild cognitive impairment
OHI: Open House Initiative
PAL: Paired Associates Learning test
p-tau: Phosphorylated tau
REN: Names recognized
REO: Occupations recognized
RLN: Names in long-term recall
RLO: Occupations in long-term recall
ROC: Receiver operating characteristic
RSN: Names in short recall
RSO: Occupations in short recall
S-FNAME: Spanish version of the Face-Name Associative Memory Exam
SD: Standard deviation
t-tau: Total tau
